# Saccadic eye movement speed is related to variations in phantom array effect visibility

**DOI:** 10.1038/s41598-023-38477-z

**Published:** 2023-07-18

**Authors:** Hyeran Kang, Chan-Su Lee, Jun-Gi Kim, Hyensou Pak

**Affiliations:** 1 Department of Electronic Engineering, Yeungnam University, Gyeongsan, 38541 Republic of Korea; 2 Department of Automotive Lighting Convergence Engineering, Yeungnam University, Gyeongsan, 38541 Republic of Korea; 3 Research Institute of Human Ecology, Yeungnam University, Gyeongsan, 38541 Republic of Korea

**Keywords:** Psychology, Human behaviour

## Abstract

The phantom array effect is one of the temporal light artefacts that can decrease performance and increase fatigue. The phantom array effect visibility shows large individual differences; however, the dominant factors that can explain these individual differences remain unclear. We investigated the relationship between saccadic eye movement speed and phantom array visibility at two different angles and four different directions of saccadic eye movement. The peak speed of saccadic eye movement and the phantom array effect visibility were measured at different modulation frequencies of the light source. Our results show that phantom array visibility increased as eye movement speed increased; the phantom array visibility was higher at a wide viewing angle with fast eye movement speed than at a narrow viewing angle. Moreover, when clustered into subgroups according to individual eye movement speed, the mean speed of the saccadic eye movement of each subgroup is related to the variations in the visibility of the phantom array effect of the subgroup. Therefore, saccadic eye movement speed is related to variations in phantom array effect visibility.

## Introduction

Temporal light modulation (TLM) is a fluctuation in the luminous quantity or spectral distribution of the output of a lighting system over time^1^. The fast response time of solid-state light sources makes the effect of TLM more prominent than that of traditional lighting sources. In many cases, TLMs, such as flicker, stroboscopic, and phantom array effect, may cause distraction in the perception of the environment^2^ and unacceptable light variation. These undesirable changes in visual perception are also called temporal light artefacts (TLA)^3^. TLA can cause a decrease in visual performance, an increase in fatigue, and health problems such as migraine^4^. Symptoms of discomfort are correlated with the visibility of the threshold frequency of the phantom array effect^5^.

The phantom array effect is the appearance of multiple images of objects lit by a temporally unstable light source as a result of eye movement^4^. The visibility of the phantom array effect can be influenced by the luminance, modulation frequency, duty cycle of the modulation^6^, and peripheral field^7^. Chromaticity, color temperature^8^, and angular width of the light source^9^ are also important factors that affect the visibility of the phantom array effect.

However, the visibility threshold frequencies of the phantom array effect exhibit large individual differences^10,11^. In addition to visual discomfort, the pattern glare sensitivity test^12^ and critical flicker frequency (CFF) are used to explain the large individual differences in the visibility of the phantom array effect. However, they provide a limited explanation for the individual differences in visibility.

In this study, we investigated the effect of saccadic eye movement speed on the visibility of the phantom array effect. The speed of saccadic eye movements differs depending on the direction of eye movement^13^. Saccadic, anti-saccadic, and smooth pursuit eye movements also show individual differences^14^. Individual differences in eye movement speed are also stable^15^. Previous studies on the visibility of the phantom array effect and eye movement speed showed a potential correlation between the threshold frequency of the visibility of the phantom array effect and eye movement speed^16,17^. However, it is difficult to ascertain individual differences in the phantom array effect visibility based on eye movement speed. In this study, we investigated the relationship between saccadic eye movement speed and the threshold frequency of the phantom array effect by analyzing the peak eye movement speed when observing the phantom array effect correctly. In addition, we analyzed subgroup characteristics of the phantom array effect visibility and the speed of saccadic eye movement to determine whether the latter can explain large variations in the former.

## Results

Phantom array effect visibility is based on the correct response rate. Saccadic eye movement speed was measured using an eye tracker, the ViewPoint EyeTracker® System (Arrington Research, USA). We filtered out many potential artifacts of the tracking data to determine the peak speed of eye movement in addition to the temporal information of the moment the experiment light turns on and off.

### Phantom array visibility at different saccadic angles (Exp. 1) and viewing directions (Exp. 2)

**Figure 1:**
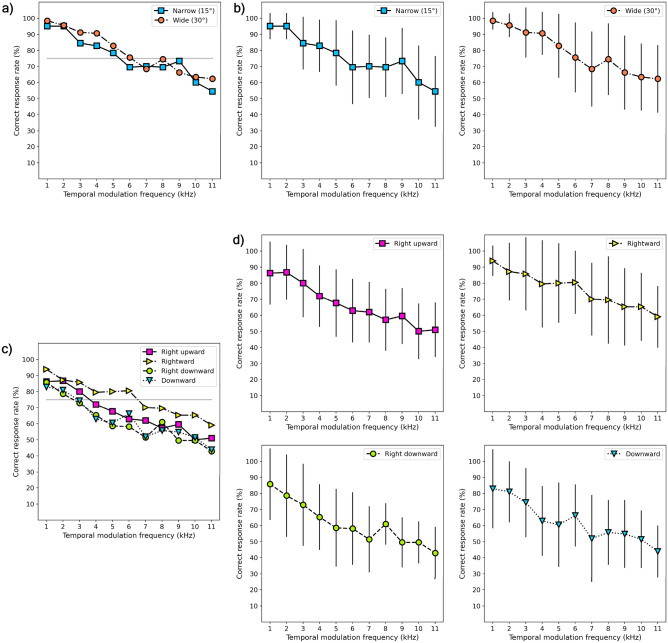
Correct response rate of the phantom array effect. (**a**) Average correct response rate of the phantom array effect at different angles of saccadic eye movement. The solid gray line indicates the 75% average correct response. (**b**) Average correct response rate of the phantom array effect at different saccadic angles: narrow saccadic angle (left) and wide saccadic angle (right). Line bar shows the standard deviation of the correct response at each frequency. (**c**) Average correct response rate of the phantom array effect at different saccadic directions. (**d**) Average correct response rate of the phantom array effect at different directions of saccadic eye movement: right upward direction (top-left), rightward direction (top-right), right downward direction (bottom-left), and downward direction (bottom-right).

The participants’ correct response rates of the phantom array effect for the saccadic angle- and direction-based experiments (Exp. 1 and 2) are described in Table 1 and Fig. 1. The frequency and correct response rate were inversely proportional, which means that phantom array visibility decreased as the modulation frequency increased. In both experiments, the visibility of the phantom array effect was evaluated based on the correct response rate of the participant. If the participant selected the correct answer in all trials, the correct response rate would be 100%, and if the response was randomly selected regardless of the visibility of the phantom array, it would be 50%. Therefore, we set the average correct response rate to 75%, which is half-way, as the visibility threshold frequency of the phantom array effect.

**Table 1:** Mean (and standard deviation) of the correct response rates of the phantom array effect of all experiments.

Experiment type	Session	Frequency										
		1 kHz	2 kHz	3 kHz	4 kHz	5 kHz	6 kHz	7 kHz	8 kHz	9 kHz	10 kHz	11 kHz
Exp. 1: Saccadic angle- based	Narrow (15°)	**89 (17)**	**90 (16)**	**80 (22)**	**76(22)**	73 (22)	67 (22)	66 (20)	67 (17)	70 (20)	57 (23)	54 (21)
	Wide (30°)	**94 (11)**	**92 (11)**	**87 (17)**	**86 (17)**	**78 (24)**	73 (21)	66 (21)	69 (25)	63 (22)	61 (20)	60 (20)
Exp. 2: Direction-based	Right upward	**86 (19)**	**87 (16)**	**80 (20)**	72 (18)	68 (20)	63 (19)	62 (18)	57 (19)	60 (17)	50 (17)	51 (16)
	Rightward	**94 (9)**	**87 (17)**	**86 (22)**	**80 (26)**	**80 (24)**	**80 (19)**	70 (22)	70 (26)	65 (23)	65 (20)	59 (18)
	Right downward	**86 (22)**	**79 (25)**	73 (25)	65 (20)	59 (23)	58 (22)	51(20)	61 (12)	50 (15)	50 (13)	43 (16)
	Downward	**83 (24)**	**81 (18)**	74 (21)	63 (21)	60 (25)	66 (19)	52 (26)	56 (19)	55 (20)	51 (17)	44 (16)

Frequency values above 75% correct response rate are marked in bold.

The correct response rate at the threshold frequency of wide saccadic angle was higher than that of narrow saccadic angle (paired t-test, t(15) = − 3.058, p = 0.003). Regarding saccadic direction, the correct response rate at the threshold frequency was high in the following order: rightward > right upward > right downward ≅ downward (ANOVA, F(3,42) = 9.951, p = 0.000).

### Eye movement speed analysis for different saccade angles (Exp. 1) and viewing directions (Exp. 2)

**Figure 2:**
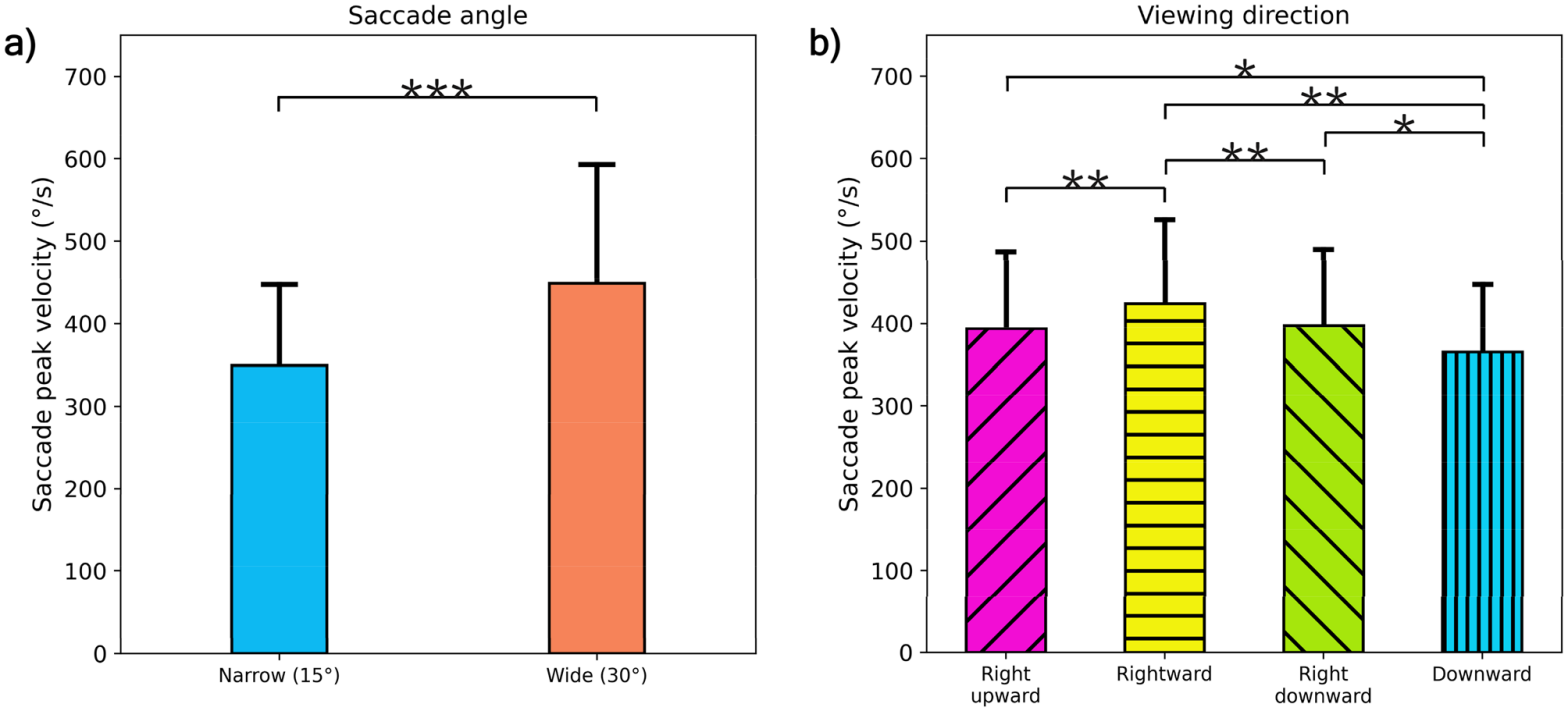
Eye movement speed analysis. (**a**) Peak velocities of the saccadic eye movement at different moving angles (Exp. 1). (**b**) Peak velocities of the saccadic eye movement at different moving directions (Exp. 2). *p < 0.05; **p < 0.01; ***p < 0.001.

The participants’ average speed based on peak saccadic eye movement when observing the phantom array effect is shown in Fig. 2. The average speeds of saccadic eye movement for the narrow and wide viewing angles are as follows: 349.31 ± 97.90°/s and 448.95 ± 143.94°/s, respectively. The speed of saccadic eye movement at a wide saccade angle was significantly faster than that of the narrow one (Fig. 2a, paired t-test, t(15) = − 4.279, p = 0.001). Average speeds of saccadic eye movement in the right upward, rightward, right downward, and downward viewing directions are 393.64 ± 93.29°/s, 414.27 ± 98.99°/s, 388.79 ± 88.94°/s, and 358.40 ± 80.85°/s, respectively. Repeated measures ANOVA showed that the difference in saccadic peak velocity according to viewing direction was statistically significant (Fig. 2b, ANOVA, F(3,42) = 37.624, p = 0.000). The speed of saccadic eye movement in the rightward direction was significantly faster than in the other directions.

### Subgroup differences in the speed of saccadic eye movement and visibility of the phantom array effect

**Figure 3:**
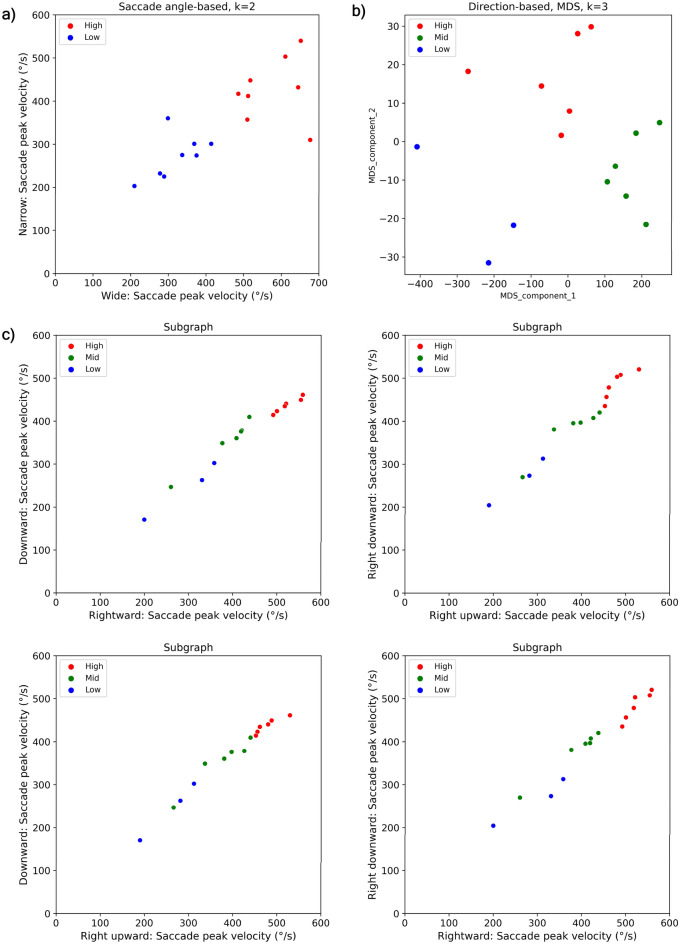
Grouping individual saccadic eye movement speed. (**a**) Scatterplots of individual saccadic speed in narrow and wide saccade angle-based experiment (Exp. 1) using k-means. (**b**) High-, middle- and low-saccadic velocity subgroups through MDS, in viewing direction-based experiment (Exp. 2). c) Scatterplots of individual saccadic speed in different direction pair (Exp. 2) using high-, middle, and low subgroups.

To analyze the individual differences in saccadic eye movements and phantom array visibility, participants were further grouped through k-means according to the speed of saccadic eye movements in each exercise condition. The K value was selected based on the elbow method^18^, and in the case of the direction-based experiment, k-means was performed after reducing it to two dimensions through multidimensional scaling (MDS)^19^. Figure 3 shows the two-dimensional embedding and clustering of the saccade angle-based (Fig. 3a) and direction-based experiments (Fig. 3b). Some scatterplot examples of individual saccadic speed in different direction pair using high-, middle, and low subgroups are shown in Fig. 3c.

Table 2 shows the subgroup mean and standard deviation of the saccadic eye movement speed (°/s) and mean threshold frequency (kHz) of phantom array visibility for different saccade angles and viewing directions.

**Table 2:** Subgroup mean (and standard deviation) of saccadic eye movement speed and mean threshold frequency of phantom array visibility at different saccade angles (Exp. 1) and viewing directions (Exp. 2) (°/s).

Experiment type	Session	Saccade peak velocity group (n = number of participants)	Saccade peak velocity (°/s)	Visibility threshold frequency (kHz)
Exp. 1: Saccade angle-based	Narrow	High (n = 8)	321.38 (65.25)	8.75 (1.98)
		Low (n = 8)	271.38 (50.72)	4.88 (3.14)
	Wide	High (n = 8)	576.38 (77.35)	9.38 (1.77)
		Low (n = 8)	427.38 (73.61)	5.25 (2.49)
Exp. 2: Direction-based	Right upward	High (n = 6)	478.35 (28.93)	5.83 (2.23)
		Mid (n = 6)	374.99 (64.44)	4.83 (3.43)
		Low (n = 3)	261.52 (63.59)	4.00 (2.00)
	Rightward	High (n = 6)	524.23 (27.35)	10.00 (1.26)
		Mid (n = 6)	387.34 (65.44)	9.50 (1.52)
		Low (n = 3)	296.39 (84.66)	4.33 (2.08)
	Right downward	High (n = 6)	483.67 (33.05)	6.83 (1.47)
		Mid (n = 6)	378.54 (54.77)	5.83 (1.94)
		Low (n = 3)	263.56 (54.91)	2.33 (1.53)
	Downward	High (n = 6)	437.15 (17.16)	6.67 (3.83)
		Mid (n = 6)	353.40 (56.10)	6.33 (2.42)
		Low (n = 3)	245.22 (67.56)	4.00 (3.61)

**Figure 4:**
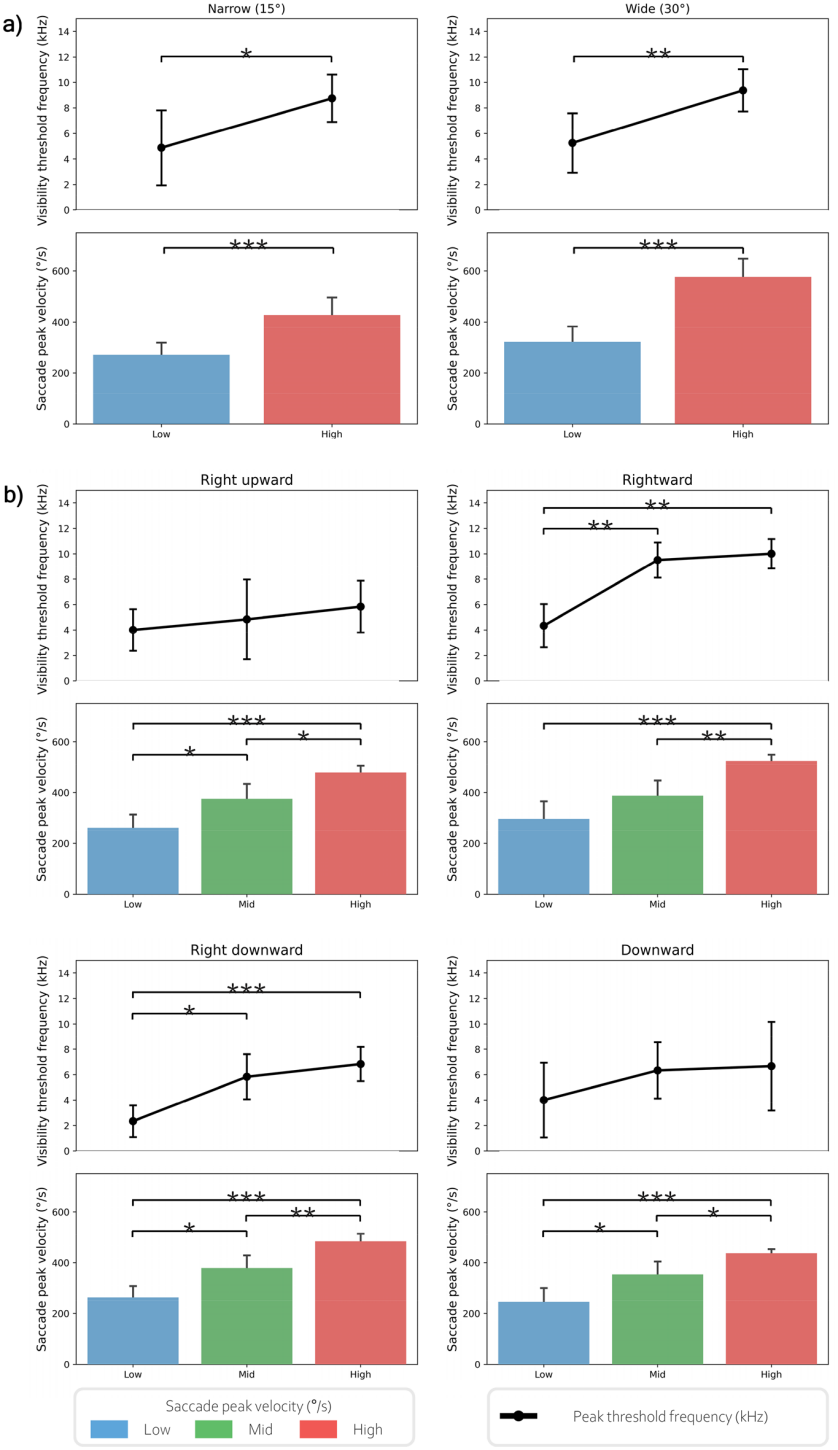
Subgroup eye movement speed and mean threshold frequency of the phantom array effect visibility. (**a**) Subgroup in saccade angle-based experiment (Exp. 1). (**b**) Subgroup in direction-based experiment (Exp. 2). *p < 0.05; **p < 0.01; ***p < 0.001.

Figure 4 shows the saccadic peak velocity and mean threshold frequency of phantom array visibility according to the subgroups classified based on saccadic peak velocity. To determine statistical significance, independent t-tests were performed for saccade angle-based experiments (Exp. 1) and one-way ANOVA for direction-based experiments (Exp. 2).

Regarding the saccade angle-based experiment (Exp. 1), the difference in saccadic peak velocity between subgroups was significant in narrow and wide angles (independent t-test, t(14) =  − 4.939, p = 0.000; t(14) =  − 7.128, p = 0.000), and the mean threshold frequency of phantom array visibility was statistically higher in the high-speed group than in the low-speed group in both narrow and wide angles (independent t-test, t(14) =  − 2.954, p = 0.010; t(14) =  − 3.818, p = 0.002).

For the direction-based experiment (Exp. 2), the saccadic velocity between the three groups was statistically significant in the right upward, rightward, right downward, and downward directions (ANOVA, F(2,14) = 17.712, p = 0.000; F(2,14) = 17.810, p = 0.000; F(2,14) = 22.767, p = 0.000; F(2,14) = 17.103, p = 0.000). The difference in the mean threshold frequency of phantom array visibility was significant in the rightward and right downward directions (ANOVA, F(2,14) = 15.160, p = 0.001; F(2,14) = 7.235, p = 0.009).

## Discussion

Saccadic eye movement speed was analyzed for two saccade angles and four viewing directions at a temporal modulation frequency of 1–11 kHz to understand the relationship between saccadic eye movement speed and phantom array effect visibility.

In the saccade angle-based experiment (Exp. 1), the threshold frequency of phantom array effect visibility was higher in the wide-angle eye movement than in the narrow one, and saccadic eye movement speed was faster in the wide-angle eye movement experiment than in the narrow one, which was statistically significant. In the direction-based experiment (Exp. 2), the threshold frequency of phantom array effect visibility was high in the order of rightward > right upward > right downward ≅ downward, and saccadic eye movement speed was high in the order of rightward > right upward > right downward > downward.

For both experiments (Exp. 1 and Exp. 2), correlation analysis was performed for phantom array effect visibility and saccadic eye movement speed. The results show that there was a positive correlation in both the saccade angle-based (Exp. 1) (r = 0.670, N = 16, p = 0.000) and direction-based (Exp. 2) (r = 0.411, N = 15, p = 0.001) experiments. The correlation analysis shows that the visibility of the phantom array effect correlates with the speed of saccadic eye movement, and both experiments show that the speed of saccadic eye movement is related to the threshold frequency of the phantom array effect by 44.9% and 16.9%, respectively. This indicates that saccadic eye movement speed can be an important factor to predict phantom array effect visibility, especially individual differences in visibility even though there seem to be other factors potentially contribute to the visibility. In addition, saccadic suppression^20,21^ might influence the phantom array visibility. However, the effect of saccadic suppression on the phantom array visibility is not known yet.

Saccadic eye movement speed varied according to the experimental conditions, even within the same participant. Phantom array effect visibility differed depending on the speed of individual saccadic eye movements. As a result of analyzing the threshold frequency by dividing it into two subgroups of low and high or three subgroups of low, mid, and high, by grouping the speed of saccadic eye movement, the threshold frequency of phantom array effect visibility was higher in the high-speed subgroup than in the low-speed subgroup in the narrow and wide saccade angle-based experiment (Exp. 1). In the direction-based experiment (Exp. 2), which used three subgroups (low, middle, and high), each subgroup showed a statistically significant difference in the threshold frequency of the visibility of the phantom array effect. However, in the right and right downward directions, each subgroup showed a statistically significant difference in the peak speed of saccadic eye movement, even though the right upward and downward directions showed an increase in the peak speed of eye movement from the low to high subgroup. Therefore, a subgroup’s saccadic eye movement speed can be useful in predicting its phantom array visibility, and large variations in saccadic eye movement speed may explain the large variation in phantom array effect visibility. Eye movement speed may not be the only factor to explain individual differences in the visibility of the phantom array effect, as it cannot explain all the variations, but it is an important factor to consider. In head-mounted display (HMD) for augmented reality (AR) and virtual reality (VR), it is important to reduce the phantom array effect^22^. Our experiment result shows that eye movement speed is a factor to be considered in the HMD development for AR and VR.

## Methods

### Experimental apparatus

**Figure 5:**
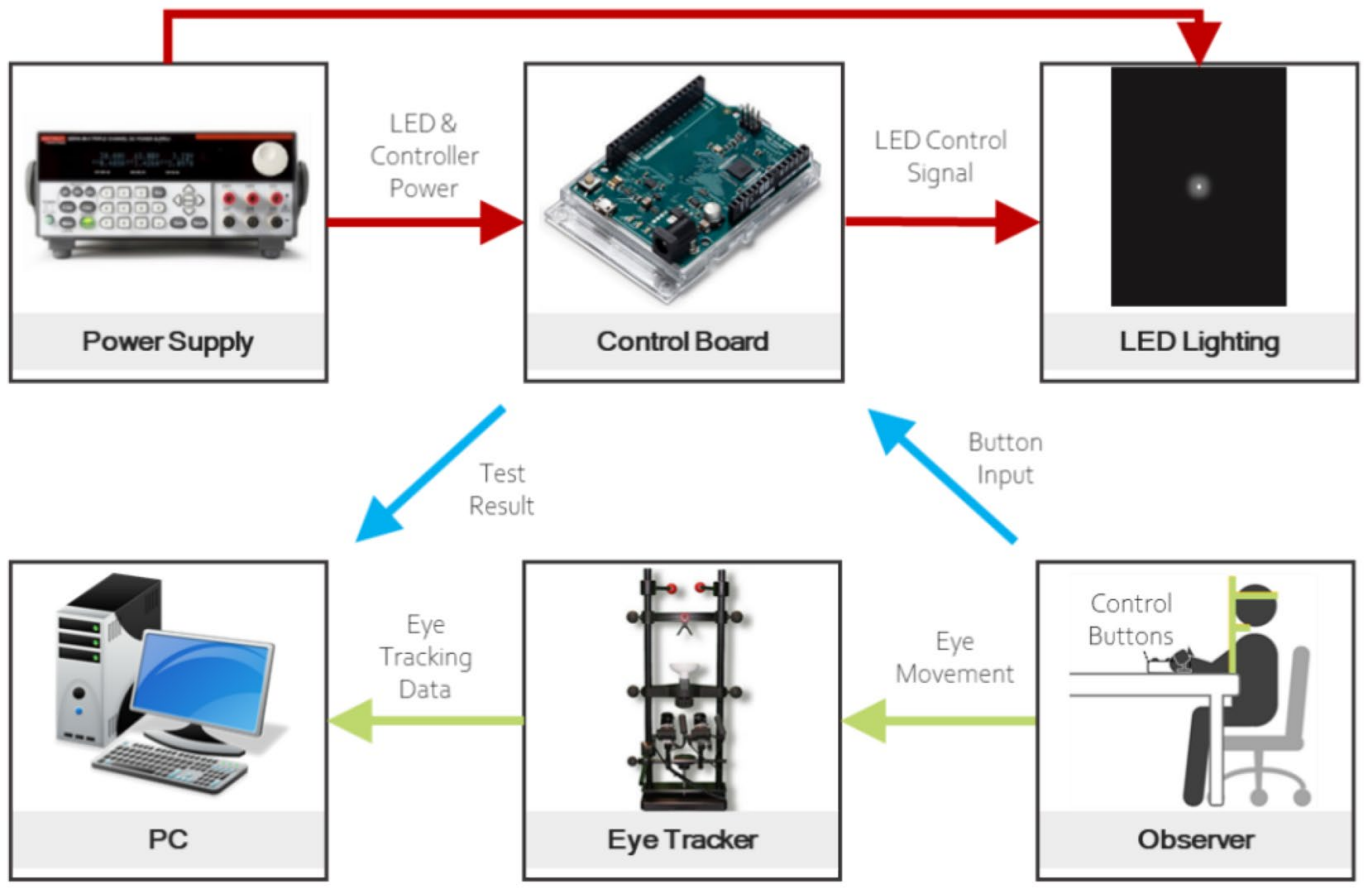
Experimental system configuration.

We developed a system comprising a DC power supply, an Arduino control board to control the lighting, LED lighting sources, a personal computer (PC) to store experimental data, an eye tracker to record participants’ eye movements, and a control button to receive data from the participant and proceed in the experiment (Fig. 5).

We used cool white LEDs as experimental stimuli. The light-emitting surface was 0.2 mm in diameter, which is 0.011°, and the central luminance was 8600 cd/m^2^, as measured by a spectroradiometer (Konica Minolta CS-2000). Pulse width modulation (PWM) frequency was controlled using an Arduino control board. The modulation frequency was randomly selected between the evaluation frequency and 60 kHz^23^. The evaluation frequency was between 1 and 11 kHz in step of 1 kHz. The PWM duty cycle was fixed at 50% with full modulation depth at a given frequency. To measure eye movement speed, the ViewPoint EyeTracker® System (Arrington Research, USA), equipped with a binocular high-resolution infrared (IR) camera, was installed in front of the participant. The eye tracker provided the location of the pupil center and its diameter in the x–y plane at 220 Hz.

### Participants

We pre-screened the recruited participants; six out of 27 recruited subjects were excluded in the pre-screening step because they could not observe the phantom array effect at 1 kHz under our experimental conditions. Twenty-one participants (11 males, 10 females) participated in the experiments. However, in some cases, participants with highly reflective glasses could not have their pupils accurately recognized at the calibration stage. As a result, we were unable to obtain data from these participants. We removed the participants whose initialization and following processing had missing data from more than one trial. Even with calibration, there were instances where the eye tracker failed to accurately recognize the pupil due to issues such as excessive tear accumulation over time. Since our experimental trial was presented 10 times for each of the 11 frequencies, we excluded trials from the analysis if there was an absence of eye movement speed in more than one out of the ten presentations. Therefore, for eye movement analysis results, data from 16 participants from the saccade angle-based experiment and 15 participants from the direction-based experiment were used.

All participants were in their twenties and were undergraduate or graduate students at Yeungnam University with no color blindness or color weakness. Written informed consent was obtained before participation. The experimental protocol was approved by the Ethics Committee (Yeungnam University, Republic of Korea). The participants received monetary compensation for their participation. All experiments were performed in accordance with relevant guidelines and regulations of the committee.

### Experiment environment

**Figure 6:**
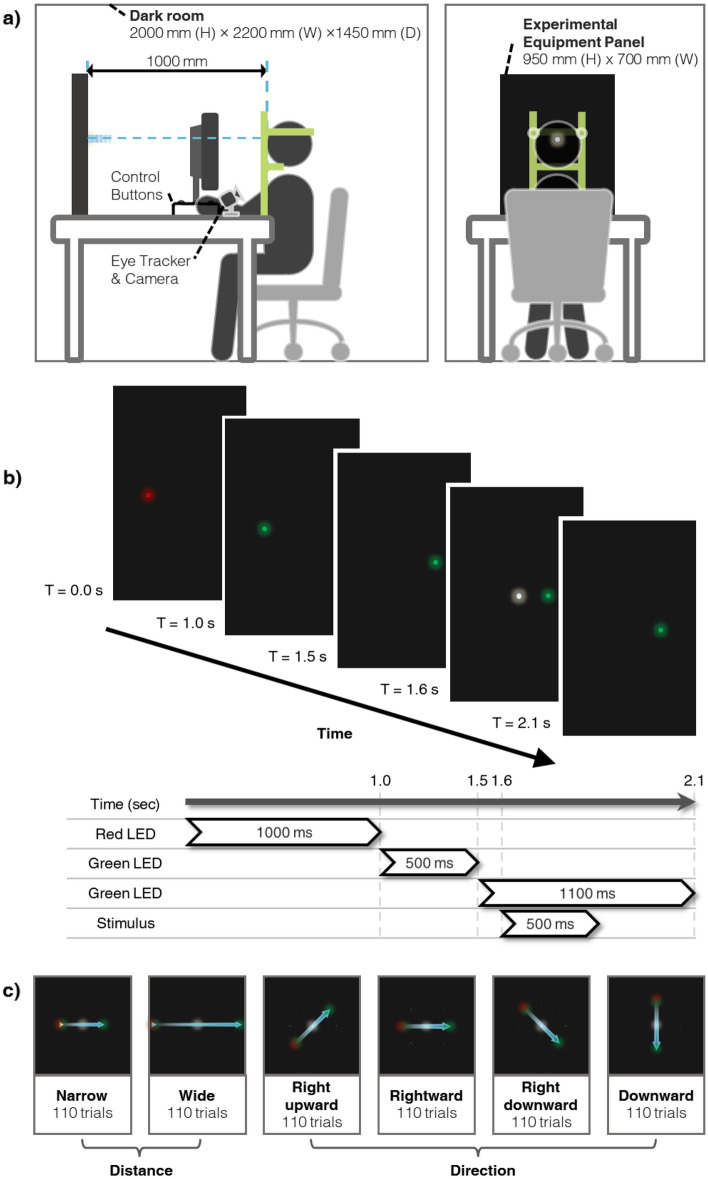
Experimental setup. (**a**) Participant location. (**b**) Timeline of an experimental trial. (**c**) Examples of task procedure. The arrow represents the direction of eye movement.

The experiment was conducted in a dark room which was 2200 × 1450 mm, with a height of 2600 mm. The experimental equipment panel, which was made of matte fomex, was 950 mm high and 700 mm wide. Participants sat 1000 mm away from the experimental stimuli, adjusted the height of a chair in the darkroom to match the eye level with the experimental stimulus on the panel, and rested their heads on a desk-mounted chin and headrest to reduce head movements. To track the participants’ eye movements, two cameras were positioned in front of them (Fig. 6a).

### Procedure

In the saccade angle-based experiment (Exp. 1), the participant observed the stimulus by moving the pupil in two different widths: 263 mm, which corresponds to 15° saccadic eye movement, for a narrow saccade angle, and 536 mm, which corresponds to 30° saccadic eye movement, for a wide saccade angle. In the direction-based experiment (Exp. 2), the stimulus was observed by moving the pupil in four directions: right upward, rightward, right downward, and downward. The viewing angle of the saccadic eye movement in the direction-based experiment was 17°. Our experimental light was presented as a small circle with a diameter of 0.2 mm, and saccadic eye movements were induced by guiding LED lights at the starting and ending points. The experimental trial was presented 10 times for each of the 11 frequencies; thus, the participants conducted 110 trials for each experimental session. As each experimental session took 30 min, the saccade angle experiment (Exp. 1) required one hour, and the direction-based experiment (Exp. 2) required two hours. In all experiments, the angle or direction of eye movement and the temporal modulation frequency order of the tasks were randomized, and participants were able to take a break whenever they wanted.

Before starting the experiment, we placed a monitor in front of each participant and removed it after calibrating the eye tracker to track the participant’s eye movement. The participants underwent dark adaptation in a dark room for 7 min and then waited while fixing their gaze on a red LED. After 1000 ms, when the experiment started, the red LED was turned off, and a green LED was turned on for 500 ms and off. After that, another green LED was turned on. The experimental stimulus was turned on and off for 500 ms after 100 ms later of turning on the second green LED. The second green LED was turned off after 1100 ms on. The participant observed the experimental stimulus while moving his/her gaze along the first green LED to the second green LED when the first green LED was off and the second green LED was on (Fig. 6b, c) (Supplementary file).

Each trial appeared as a pair, and the participant pressed a button to indicate which of the two trials was the phantom array effect observed. If they did not observe the phantom array effect in either trial, they had to guess and choose one.

## Supplementary Information

Supplementary Information.
