# Supplementary material for: Saccadic eye movement speed is related to variations in phantom array effect visibility
Source: Sci Rep. 2023 Jul 18;13:11576. doi: 10.1038/s41598-023-38477-z (PMC10354087; doi:10.1038/s41598-023-38477-z)
Supplement: Supplementary file 1 — Supplementary Information. [file 41598_2023_38477_MOESM1_ESM.pdf]

Distance-based experiment result(Raw data):

Participant number			P1	P2	S3	P4	P5	P6	P7	P8	P9	P10	P11	P12	P13	P14	P15	P16
Saccade peak velocity (°/s)	Narrow		203.44	231.53	224.99	359.57	274.6	300.68	274.27	301.09	417.4	356.74	411.85	448.14	503.14	432.11	539.76	309.58
	Wide		210.27	278.04	289.09	299.03	337.43	369.47	374.52	414.2	486.35	509.65	512.34	517.84	610.95	644.88	652.36	676.85
Correct response rate of the phantom array effect (%)	Narrow	1kHz	100	90	90	100	90	100	90	100	70	100	100	90	100	100	90	100
		2kHz	80	90	100	100	90	100	80	100	100	100	100	90	80	100	100	100
		3kHz	70	50	80	50	90	100	80	100	90	90	100	100	80	100	90	100
		4kHz	80	60	60	70	80	100	70	90	80	100	100	70	100	100	70	100
		5kHz	70	30	80	70	70	100	50	100	90	60	90	90	80	100	70	100
		6kHz	40	30	60	50	70	100	60	80	40	50	100	90	90	100	60	80
		7kHz	50	60	60	50	70	100	50	80	70	30	80	70	70	100	60	100
		8kHz	70	50	60	50	70	100	40	80	80	50	50	70	60	100	80	90
		9kHz	50	70	50	50	50	100	70	60	90	40	80	90	80	100	90	90
		10kHz	60	20	40	70	50	90	40	50	50	40	50	50	70	100	80	80
		11kHz	40	60	30	70	40	90	70	20	40	70	60	60	50	70	40	70
	Wide	1kHz	100	100	100	100	80	100	90	100	100	100	100	100	100	100	100	100
		2kHz	90	100	100	100	90	100	90	100	90	100	100	80	100	100	100	100
		3kHz	80	40	80	100	100	100	100	100	90	100	100	80	90	100	100	100
		4kHz	90	80	80	100	80	100	50	100	90	100	100	80	90	100	100	100
		5kHz	60	50	60	80	90	100	60	90	100	100	100	90	90	100	90	90
		6kHz	40	40	70	50	70	100	70	80	100	80	100	70	100	100	60	80
		7kHz	30	40	50	30	60	100	50	70	70	70	100	80	70	100	80	90
		8kHz	70	60	40	50	70	100	60	50	70	50	90	50	100	100	100	100
		9kHz	40	60	40	70	60	100	30	50	70	60	50	90	60	100	100	90
		10kHz	70	20	70	60	30	100	60	40	80	60	50	70	80	100	60	60
		11kHz	50	70	30	50	60	100	30	60	50	60	70	50	80	100	60	90

Direction-based experiment result(Raw data):

Participant number			P1	P2	S3	P4	P5	P6	P7	P8	P9	P10	P11	P12	P13	P14	P15
Saccade peak velocity (°/s)	Right upward		312.53	480.52	452.88	190.27	461.77	456.40	530.04	337.43	281.76	381.08	440.76	266.34	488.50	397.74	426.59
	Right		408.83	492.10	437.98	199.95	558.76	518.32	554.41	358.46	330.75	376.76	500.57	260.34	521.23	419.21	420.90
	Right downward		395.44	435.25	407.66	204.39	478.44	507.90	520.85	312.86	273.44	380.82	456.43	270.03	503.15	396.87	420.39
	Downward		409.59	434.56	449.34	170.63	360.44	414.28	440.31	302.30	246.81	376.21	348.86	262.74	461.33	378.47	423.05
Correct response rate of the phantom array effect (%)	Right upward	1kHz	100	90	100	90	90	90	90	100	100	100	100	70	100	100	100
		2kHz	100	100	100	90	100	100	100	90	90	100	90	80	100	100	100
		3kHz	100	60	100	50	100	100	90	100	100	90	100	70	100	100	60
		4kHz	80	60	90	80	70	100	50	90	80	80	70	60	100	100	70
		5kHz	100	80	90	60	80	80	60	80	80	70	50	70	100	80	40
		6kHz	100	70	60	40	60	90	50	70	90	80	60	60	100	60	50
		7kHz	80	50	70	40	70	70	60	60	70	30	60	50	100	100	50
		8kHz	40	60	50	20	70	80	50	60	60	60	60	70	80	100	50
		9kHz	100	50	30	50	70	50	60	50	70	50	70	40	80	80	50
		10kHz	60	60	50	70	30	70	70	20	20	30	60	50	70	60	50
		11kHz	60	40	60	60	60	40	10	50	40	30	30	40	70	80	40
	Rightward	1kHz	100	100	100	90	100	100	100	100	100	100	100	80	90	100	100
		2kHz	100	100	100	100	100	80	100	100	100	80	100	80	100	100	100
		3kHz	100	100	100	70	100	90	100	100	100	100	100	100	100	100	100
		4kHz	100	80	100	50	100	90	100	100	100	100	100	90	100	100	100
		5kHz	100	100	100	70	100	100	100	90	90	90	100	60	100	100	100
		6kHz	100	70	100	50	90	80	100	50	90	70	100	60	100	100	100
		7kHz	100	60	70	30	80	80	80	60	60	100	90	40	100	100	100
		8kHz	100	80	100	50	90	90	50	60	60	40	90	40	100	80	70
		9kHz	100	40	90	50	90	70	60	40	70	60	100	80	100	100	40
		10kHz	100	50	60	70	70	60	100	50	60	50	90	50	100	80	80
		11kHz	80	30	50	40	50	80	80	70	60	70	70	40	80	100	60
	Right downward	1kHz	100	100	100	90	90	100	100	100	100	80	100	100	100	100	100
		2kHz	100	80	90	80	100	90	90	20	90	100	100	50	100	100	100
		3kHz	100	60	90	60	100	90	80	60	70	100	100	100	100	80	80
		4kHz	80	40	80	80	90	90	80	40	30	50	90	60	90	80	80
		5kHz	100	50	60	60	100	80	70	60	30	50	80	10	100	40	60
		6kHz	80	80	70	40	70	80	80	60	40	20	90	70	100	60	50
		7kHz	40	40	80	30	60	70	50	40	60	60	60	90	80	60	30
		8kHz	60	70	70	70	60	50	80	60	50	70	70	50	50	80	70
		9kHz	40	60	30	30	40	50	40	50	50	30	90	60	40	60	40
		10kHz	40	60	40	60	40	40	70	70	50	60	60	50	30	40	60
		11kHz	20	60	40	40	60	10	60	30	40	40	60	30	50	40	70
	Downward	1kHz	100	100	100	90	100	100	100	100	100	70	100	80	100	100	100
		2kHz	100	80	90	70	80	90	90	90	100	100	100	60	100	100	80
		3kHz	80	40	80	70	90	90	80	80	100	100	100	70	100	100	60
		4kHz	60	40	50	50	90	50	80	50	100	50	90	50	100	80	40
		5kHz	60	80	70	50	70	80	90	30	90	70	90	50	100	100	30
		6kHz	80	50	60	40	70	60	60	50	100	50	90	70	100	100	70
		7kHz	20	50	20	50	50	60	30	50	100	30	100	30	100	60	50
		8kHz	60	60	50	80	60	30	60	60	80	70	80	70	90	40	50
		9kHz	80	90	30	40	60	60	50	50	50	50	50	50	100	0	40
		10kHz	0	60	50	60	50	50	70	70	50	60	60	30	80	60	60
		11kHz	40	40	50	50	10	80	40	50	50	50	50	40	30	20	60
